# Observation of the density dependence of the closed-channel fraction of a ^6^Li superfluid

**DOI:** 10.1093/nsr/nwab226

**Published:** 2021-12-23

**Authors:** Xiang-Pei Liu, Xing-Can Yao, Hao-Ze Chen, Xiao-Qiong Wang, Yu-Xuan Wang, Yu-Ao Chen, Qijin Chen, Kathryn Levin, Jian-Wei Pan

**Affiliations:** Hefei National Laboratory for Physical Sciences at the Microscale and Department of Modern Physics, University of Science and Technology of China, Hefei 230026, China; Shanghai Branch, CAS Center for Excellence in Quantum Information and Quantum Physics, University of Science and Technology of China, Shanghai 201315, China; Shanghai Research Center for Quantum Sciences, Shanghai 201315, China; Hefei National Laboratory for Physical Sciences at the Microscale and Department of Modern Physics, University of Science and Technology of China, Hefei 230026, China; Shanghai Branch, CAS Center for Excellence in Quantum Information and Quantum Physics, University of Science and Technology of China, Shanghai 201315, China; Shanghai Research Center for Quantum Sciences, Shanghai 201315, China; Hefei National Laboratory for Physical Sciences at the Microscale and Department of Modern Physics, University of Science and Technology of China, Hefei 230026, China; Shanghai Branch, CAS Center for Excellence in Quantum Information and Quantum Physics, University of Science and Technology of China, Shanghai 201315, China; Shanghai Research Center for Quantum Sciences, Shanghai 201315, China; Hefei National Laboratory for Physical Sciences at the Microscale and Department of Modern Physics, University of Science and Technology of China, Hefei 230026, China; Shanghai Branch, CAS Center for Excellence in Quantum Information and Quantum Physics, University of Science and Technology of China, Shanghai 201315, China; Shanghai Research Center for Quantum Sciences, Shanghai 201315, China; Hefei National Laboratory for Physical Sciences at the Microscale and Department of Modern Physics, University of Science and Technology of China, Hefei 230026, China; Shanghai Branch, CAS Center for Excellence in Quantum Information and Quantum Physics, University of Science and Technology of China, Shanghai 201315, China; Shanghai Research Center for Quantum Sciences, Shanghai 201315, China; Hefei National Laboratory for Physical Sciences at the Microscale and Department of Modern Physics, University of Science and Technology of China, Hefei 230026, China; Shanghai Branch, CAS Center for Excellence in Quantum Information and Quantum Physics, University of Science and Technology of China, Shanghai 201315, China; Shanghai Research Center for Quantum Sciences, Shanghai 201315, China; Hefei National Laboratory for Physical Sciences at the Microscale and Department of Modern Physics, University of Science and Technology of China, Hefei 230026, China; Shanghai Branch, CAS Center for Excellence in Quantum Information and Quantum Physics, University of Science and Technology of China, Shanghai 201315, China; Shanghai Research Center for Quantum Sciences, Shanghai 201315, China; James Franck Institute, University of Chicago, Chicago, IL 60637, USA; Hefei National Laboratory for Physical Sciences at the Microscale and Department of Modern Physics, University of Science and Technology of China, Hefei 230026, China; Shanghai Branch, CAS Center for Excellence in Quantum Information and Quantum Physics, University of Science and Technology of China, Shanghai 201315, China; Shanghai Research Center for Quantum Sciences, Shanghai 201315, China

**Keywords:** closed-channel fraction, density dependence, strongly interacting Fermi gas, many-body effect

## Abstract

Atomic Fermi gases provide an ideal platform for studying pairing and superfluid physics, using a Feshbach resonance between closed-channel molecular states and open-channel scattering states. Of particular interest is the strongly interacting regime. We show that the closed-channel fraction ${Z_{{\rm{cc}}}}$ provides an effective probe for important many-body interacting effects, especially through its density dependence, which is absent from two-body theoretical predictions. Here we measure ${Z_{{\rm{cc}}}}$ as a function of interaction strength and the Fermi temperature ${T_{\rm{F}}}$ in a trapped ^6^Li superfluid throughout the entire Bardeen-Cooper-Schrieffer–Bose-Einstein-condensate crossover, in quantitative agreement with theory when important thermal contributions outside the superfluid core are taken into account. Away from the deep-BEC regime, the fraction ${Z_{\rm cc}}$ is sensitive to ${{{T}}_F}$. In particular, our data show ${Z_{{\rm{cc}}}} \propto T_{\rm{F}}^\alpha $ with $\alpha = {\rm{1/2}}$ at unitarity, in quantitative agreement with calculations of a two-channel pairing fluctuation theory, and $\alpha $ increases rapidly into the BCS regime, reflecting many-body interaction effects as predicted.

## Teaser

The closed-channel fraction in a Fermi superfluid of ^6^Li atoms has been shown to exhibit a strong systematic density dependence, which reveals important many-body interaction effects, as theoretically predicted.

## INTRODUCTION

With a Feshbach resonance (FR), atomic Fermi gases [1] provide an ideal platform for studying pairing and superfluid physics. Of particular interest is the crossover [2,3] from a Bardeen-Cooper-Schrieffer (BCS) state of Cooper pairs to a Bose-Einstein condensate (BEC) of molecular dimers [4–11]. Unlike in a one-channel model, where the effect of FR is oversimplified into a tunable non-retarded pairing interaction strength, the rich and important physics of FR can be described by a two-channel model [3], in which open-channel atom pairs are linearly superimposed with closed-channel molecules [12–14], and are further dressed with many-body interactions. These ‘dressed molecules’ can be represented symbolically as ${{\rm{\Psi }}_{{\rm{dressed}}}} = \sqrt Z {{\rm{\Psi }}_{{\rm{closed}}}} + \sqrt {1 - Z} {{\rm{\Psi }}_{{\rm{open}}}}$, where $Z$ reflects the closed-channel fraction within the dressed molecules. While the superposition reflects two-body physics, the underlying dressing reflects important many-body physics. Indeed, many important physical quantities, such as the superfluid excitation gap [15–17] and Tan's contact [18–22], can be associated with the closed-channel fraction. The measurement of how the closed-channel fraction evolves with interaction, density and temperature can thus provide crucial information on many-body interaction effects and serve as a benchmark to test various many-body theories.

Experimentally [23], it is more convenient to measure the closed-channel fraction ${Z_{\rm cc}}$ of the entire Fermi gas. One could obtain $Z$ by dividing ${Z_{{\rm{cc}}}}$ with the pair fraction [3], which is unity in the deep-BEC regime but becomes small in the BCS regime. In the simple two-body theory [24], ${Z_{{\rm{cc}}}}$ decreases from 1 in the BEC limit to 0 at unitarity, beyond which the attractive interaction becomes too weak to support bound molecules. When it is large, ${Z_{{\rm{cc}}}}$ can be determined by the derivative of the binding energy of dressed molecules with respect to the magnetic field $B$ [25]. However, this method does not work when ${Z_{{\rm{cc}}}}$ is small, due to limited experimental resolution as well as the breakdown of the two-body theory in the unitary and BCS regimes. By driving transitions between the dressed molecules and molecules in excited states with a resonant laser, ${Z_{{\rm{cc}}}}$ has been previously measured in the BCS–BEC crossover of a ^6^Li superfluid [23]. While ${Z_{{\rm{cc}}}}$ decreased from the BEC to the unitary regime, a non-vanishing and smoothly varying ${Z_{{\rm{cc}}}}$ was observed across unitarity into the BCS regime [23]. Despite the fact that the quantity ${Z_{{\rm{cc}}}}$ was extracted assuming an exponential decay of the remaining atom number $N$ versus laser probing time ${{t}}$ based on the two-body theory, this observation indicates that the many-body effect must be present in the BCS regime. Indeed, ${Z_{{\rm{cc}}}}$ was considered to be related to the square of excitation gap in this regime and slight deviation from exponential loss for $N$ was noticed [23]. Nonetheless, due to limited signal-to-noise ratio and large error bars in atomic numbers, a clear dependence of ${Z_{{\rm{cc}}}}$ on the particle number $N$, which manifests the many-body effects, was not observed.

This experiment has been addressed to various degrees by many-body-based two-channel models [14,16,19,26]. It is shown that ${Z_{{\rm{cc}}}}$ depends not only on the scattering length but also on the Fermi temperature ${T_{\rm{F}}}$ of the system [16,26], unlike in a previous experiment [23], which reported a single unique value of ${Z_{{\rm{cc}}}}$ for a given scattering length. In particular, a universal relation of ${Z_{{\rm{cc}}}} \propto \sqrt {{T_{\rm{F}}}} $ at unitarity is predicted. A direct consequence of these predictions is that, although very weak, ${Z_{{\rm{cc}}}}$ is a function of the particle number $N$, via ${Z_{{\rm{cc}}}} \propto T_{\rm{F}}^\alpha \propto {N^{\alpha /3}}$ with $\alpha = {\rm{1/2}}$ at unitarity and $\alpha > {\rm{1/2}}$ in the BCS regime, resulting in a power-law decay of atom number as a function of laser probe time. Importantly, in the $N \to {\rm{0}}$ limit, ${Z_{{\rm{cc}}}}$ vanishes both at unitarity and in the BCS regime, consistent with the two-body result.

In this paper, we report on precision measurements of the closed-channel fraction ${Z_{{\rm{cc}}}}$ as a function of magnetic field and Fermi temperature ${T_{\rm{F}}}$ in ^6^Li superfluid at low ${\rm{T}}$ with optical molecular spectroscopy, and provide unambiguous evidence that ${Z_{{\rm{cc}}}}$ is governed by many-body physics. We emphasize that a concrete relation between ${Z_{{\rm{cc}}}}$ and density can be used to extract other physical properties and to test various theories, and is thus much more important than simply knowing ${Z_{{\rm{cc}}}} \ne {\rm{0}}$ in the BCS regime. Due to the smallness of ${Z_{{\rm{cc}}}}$ [23], precise control of experimental parameters is needed in order to unravel the many-body interaction effect. Indeed, we find that, due to the weak dependence on particle number, many-body effects can be easily buried in noise, as was the case in ref. [23]. With advanced laser cooling techniques, we are able to produce a ^6^Li superfluid with large atom number and very low temperature, which greatly improve the signal-to-noise ratio of the measurements. Moreover, to reduce systematic errors, the Rabi frequency of the molecular transition is calibrated with the well-known ${Z_{{\rm{cc}}}}$ values in the BEC regime. With these improved techniques and calibrations, in the unitary and BCS regimes, power-law fittings that account for the many-body effects are in good accordance with the experimental data, while obvious deviations are found for two-body-theory-based exponential fittings, especially at high ${\rm{B}}$. At unitarity, the universal relation ${Z_{{\rm{cc}}}} = \eta \sqrt {{T_{\rm{F}}}} $ has been revealed, with $\eta = {\rm{0}}{\rm{.074}}( {{\rm{12}}} ){\rm{\ }}{{\rm{K}}^{{\rm{ - 1/2}}}}$, in quantitative agreement with the theoretical prediction [16,26] (note here ${\rm{K}}$ denotes Kelvin). At a higher field $B = {\rm{925}}$ G, in the near-BCS regime, we find a power-law exponent much larger than 1/2, in quantitative agreement with predictions as well. This higher exponent means a more sensitive dependence of ${Z_{{\rm{cc}}}}$ on ${T_{\rm{F}}}$ and hence a stronger many-body effect. Furthermore, a proper treatment of thermal contributions of the closed-channel molecules is of crucial importance. The data and theory in the BCS regime can be brought into quantitative agreement by taking into account contributions of thermal non-condensed closed-channel molecules outside the superfluid core in the trap.

## RESULTS AND DISCUSSION

The experimental procedure for producing ^6^Li superfluid has been described in our previous works [27,28]. The superfluid of ${\rm{3}}{\rm{.0(1)\ \times \ 1}}{{\rm{0}}^{\rm{6}}}$ ^6^Li atoms at $T/{T_{\rm{F}}}{\rm{\ }} = {\rm{\ 0}}{\rm{.05(1)}}$ is confined in an oblate harmonic optical dipole trap (wavelength of 1064 nm, ${\rm{1/}}{{{e}}^{\rm{2}}}$ horizontal (vertical) radius of 200 $\mu {\rm{m}}$ (48 $\mu {\rm{m}}$)). The radial confinement is mainly optical with horizontal and vertical trap frequencies being ${\rm{2\pi \ \times \ 53}}{\rm{.7(3)}}$ Hz and ${\rm{2\pi \ \times \ 205}}{\rm{.3(5)}}$ Hz, respectively. The axial confinement is mainly provided by the magnetic field curvature with a trap frequency of ${\rm{2\pi \ \times \ 16}}{\rm{.8(1)}}$ Hz at 832 G. The method for probing the closed-channel fraction ${{\rm{Z}}_{{\rm{cc}}}}$ is similar to ref. [23], where a resonant laser transition is used to pump the closed-channel molecules into an excited singlet molecular state. Here, the transition is ${\rm{X}}{}_{\rm{\ }}^{\rm{1}}{\rm{\Sigma }}_g^ + ( {\nu = {\rm{38}}} ) \to {\rm{A}}{}_{\rm{\ }}^{\rm{1}}{\rm{\Sigma }}_u^ + ( {\nu ^{\prime} = {\rm{68}}} )$, since it possesses the largest Franck-Condon wave-function overlap. Due to Rabi oscillation and spontaneous emission loss, the number of dressed molecules decreases at rate ${\rm{\Gamma }} = {Z_{{\rm{cc}}}}{\rm{\Omega }}_{\rm{m}}^{\rm{2}}/{\gamma _{\rm{m}}}$, where ${\Omega _{\rm{m}}}$ is the Rabi frequency of the transition and ${\gamma _{\rm{m}}}$ is the linewidth of the excited molecular state. We mention that the ${\rm{\Gamma }}$ expression is valid provided ${\rm{\Omega }}_{\rm{m}}^{\rm{2}}/\gamma _{\rm{m}}^{\rm{2}} \ll {\rm{1}}$, which is easily satisfied in our experiment, and has been further verified by varying the probe laser intensity. Therefore, by recording the remaining atom number versus probing time, the fraction ${Z_{{\rm{cc}}}}$ can be extracted with given ${\rm{\Omega }}_{\rm{m}}^{\rm{2}}/{\gamma _{\rm{m}}}$.

Previously, ${\rm{\Omega }}_{\rm{m}}^{\rm{2}}/{\gamma _{\rm{m}}}$ was directly calculated based on the theoretical knowledge of molecular optical transition and the measured laser beam parameters. This method relies on precise measurement of laser power $P$ and beam waist ${\omega _{\rm{0}}}$ at the position of the atoms, which is very difficult to achieve in practice [29]. Moreover, the calculated wave-function overlap between the ground and excited molecular states is based on some theoretical assumptions regarding molecular potentials. Thus, the combination of these problems limits the accuracy of the obtained ${\rm{\Omega }}_{\rm{m}}^{\rm{2}}/{\gamma _{\rm{m}}}$ to a few ten percent. To solve this problem, the ratio ${\rm{\Omega }}_{\rm{m}}^{\rm{2}}/{\gamma _{\rm{m}}}$ is determined by calibrating the measured ${Z_{{\rm{cc}}}}$ against theory in the well-understood deep-BEC regime, where good agreement between two-body and many-body theories has been achieved [16]. Indeed, in this regime, ${Z_{{\rm{cc}}}}$ becomes independent of ${T_{\rm{F}}}$ [16], so that the molecule number will decay exponentially with a decay constant ${\rm{\Gamma }}$ upon laser exposure. Therefore, ${\rm{\Omega }}_{\rm{m}}^{\rm{2}}/{\gamma _{\rm{m}}}$ can be derived by linearly fitting a series of theoretically calculated ${Z_{{\rm{cc}}}}$ and experimentally measured ${\rm{\Gamma }}$ at different magnetic fields.

**Figure 1.**
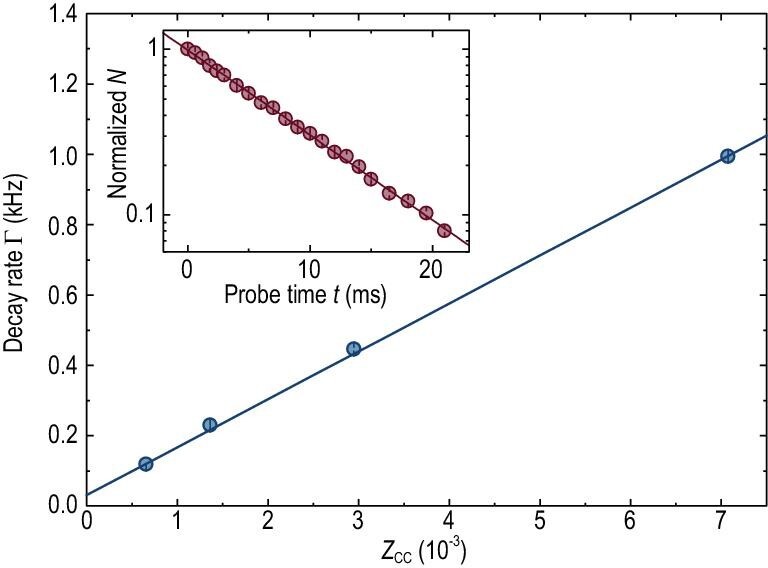
Calibration of the molecular transition parameter ${\rm{\Omega }}_{\rm{m}}^{\rm{2}}/{\gamma _{\rm{m}}}$. Shown is the decay rate ${\rm{\Gamma }}$, measured at 748, 722, 696 and 670 G, corresponding to the data points from left to right, respectively, versus theoretically calculated ${Z_{{\rm{cc}}}}$. Each data point is averaged over three measurements with error bars given by standard deviation. The slope of the linear fit yields ${\rm{\Omega }}_{\rm{m}}^{\rm{2}}/{\gamma _{\rm{m}}}$. The decay rate ${\rm{\Gamma }}$ was obtained by exponential fitting of the remaining atoms $N( t )$ (normalized at $t{\rm{\ }} = {\rm{\ 0}}$) as a function of the probe time $t$ (red line), as shown in the inset for 748 G.

After preparing the ^6^Li superfluid at 832 G, the field is linearly ramped to the desired value in 100 ms and held for another 100 ms for equilibration before the molecular probing. The probe laser (laser power of 20 $\mu {\rm{W}}$) is frequency-locked to an ultra- stable Fabry-Perot cavity with its power stabilized. The achieved long-term stability of frequency and power are 500 kHz and 0.1%, respectively, which greatly suppress the systematic errors. The measurements in the BEC regime are performed at 670, 696, 722 and 748 G, respectively. Note that the ${{1/e}}$ depletion time of milliseconds is far shorter than the molecule lifetime of more than 10 s, thus the background molecule loss is negligible. As an example, the inset of Fig. 1 shows the remaining atom number as a function of probing time at 748 G, which yields ${\rm{\Gamma }} = {\rm{118}}{\rm{.1(9)}}$ Hz. Plotted in Fig. 1 is the linear fitting to ${\rm{\Gamma }}$ versus theoretical ${Z_{{\rm{cc}}}}$ values at these magnetic fields, yielding a slope value of ${\rm{\Omega }}_{\rm{m}}^{\rm{2}}/{\gamma _{\rm{m}}} = {\rm{136(1)}}$ kHz. The high-quality fitting curve demonstrates not only the stability of our experimental set-up, but also the reliability of obtained ${\rm{\Omega }}_{\rm{m}}^{\rm{2}}/{\gamma _{\rm{m}}}$.

Before we move on to the measurements in the unitary and BCS regimes, we summarize the theory predictions [16] that lay the foundation for our data analysis. The theory includes, from the start, the two-channel Feshbach physics, as described by the Hamiltonian in ref. [30]. At $T = {\rm{0}}$ in a homogeneous Fermi gas, the superfluid order parameter ${\rm{\tilde{\Delta }}}$ associated with condensed dressed molecules has two contributions, ${\phi _{\rm{m}}}$ from the closed channel and $\Delta $ from the open channel, as ${\rm{\tilde{\Delta }}} = \Delta - g{\phi _{\rm{m}}}$, where ${n_{{\rm{b0}}}} = {\rm{\ }}\phi _{\rm{m}}^{\rm{2}}$ is the number of closed-channel molecules, and $g$ is the inter-channel coupling [3]. Note that Cooper pairing in the BCS regime is purely a many-body effect, and it is due to this linear combination that the closed-channel molecules acquire a finite fraction in the BCS regime. In the end, we have ${n_{{\rm{b}}0}} = {Z_g}{{\rm{\tilde{\Delta }}}^{\rm{2}}}$, where the coefficient ${Z_g}$ can be calculated using experimental parameters and the fermionic chemical potential. Using a local density approximation, the trap-averaged closed-channel fraction ${Z_{{\rm{cc}}}}$, as measured here, is thus given by ${Z_{{\rm{cc}}}} = {\rm{2}}{N_{{\rm{b0}}}}/N$ at low $T$, where ${N_{{\rm{b}}0}}$ and $N$ are trap integral of local ${n_{{\rm{b0}}}}( r )$ and overall atom density ${{n}}( {{r}} )$, respectively. The theory predicts that ${Z_{{\rm{cc}}}} = \eta \sqrt {{T_{\rm{F}}}} $ at unitarity (832 G), with $\eta = {\rm{0}}{\rm{.066\ }}{{\rm{K}}^{{\rm{ - 1/2}}}}$, where ${\rm{1}}/{k_{\rm{F}}}a = {\rm{0}}$, holds for the whole trap. Here, ${T_{\rm{F}}} = \hbar \bar{\omega }{( {{\rm{3}}N} )^{{\rm{1/3}}}}/{k_{\rm{B}}}$, where ${{N}}$ is the atom number and ${\rm{\bar{\omega }}}$ is the geometric average trap frequency. Away from unitarity, the local ${\rm{1}}/{k_{\rm{F}}}a$ become inhomogeneous across the trap, so that ${Z_{{\rm{cc}}}}$ as a function of ${T_{\rm{F}}}$ can only be calculated numerically, as shown in Fig. 3 of ref. [16]. Here we have recalculated ${Z_{{\rm{cc}}}}$ using the most up-to-date resonance parameters [31]. A log-log plot of ${Z_{{\rm{cc}}}}$ versus ${T_{\rm{F}}}$, presented in Fig. S1, indicates that within the range of ${T_{\rm{F}}}$ for our experimental data, it can be reasonably approximated with a power-law dependence, ${Z_{{\rm{cc}}}} \propto T_{\rm{F}}^\alpha $, with $\alpha > {\rm{1/2}}$ in the BCS regime.

For both the unitary and BCS regimes, we shall write ${Z_{{\rm{cc}}}} = \eta T_{\rm{F}}^\alpha $. Substituting this into the decay equation, one obtains:

(1) \begin{equation*} \begin{array}{*{20}{c}}{\frac{{\dot{N}}}{N} = - {Z_{{\rm{cc}}}}\frac{{{\rm{\Omega }}_{\rm{m}}^2}}{{{\gamma _{\rm{m}}}}} = - \beta {N^{\alpha /{\rm{3}}}},} \end{array} \end{equation*}

where $\beta = \eta {( {{{\rm{3}}^{{\rm{1/3}}}}\hbar \bar{\omega }/{k_{\rm{B}}}} )^{\rm{\alpha }}}{\rm{\Omega }}_{\rm{m}}^{\rm{2}}/{\gamma _{\rm{m}}}$. This leads to a power-law decay:

(2) \begin{equation*}\begin{array}{*{20}{c}} {N( t ) = \rho {{\left( {t + {\rm{\Delta }}t} \right)}^{ - c}},} \end{array} \end{equation*}

where $\alpha = {\rm{3}}/c$ and $\beta = c/{\rho ^{\alpha /{\rm{3}}}}$. Then, the parameters $\eta $ and $\alpha $ can be acquired by fitting the experimental data with Equation (2).

**Figure 2.**
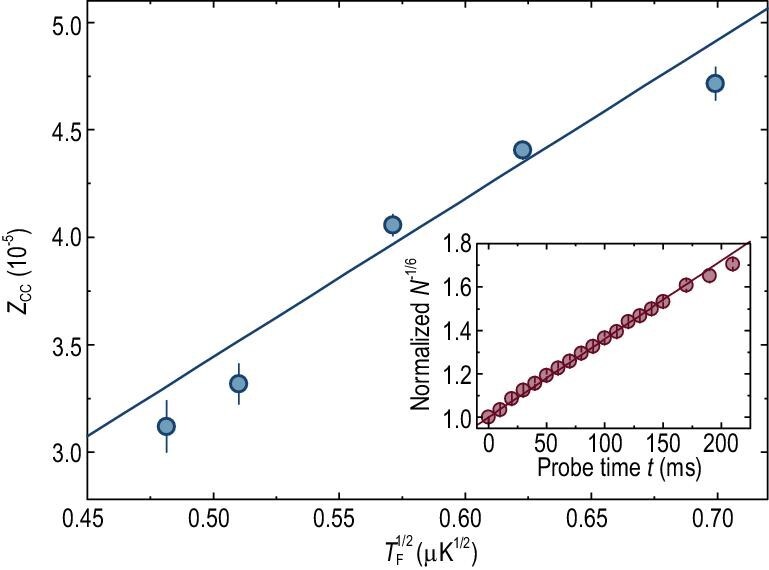
Measured ${Z_{{\rm{cc}}}}$ as a function of $T_{\rm{F}}^{{\rm{1/2}}}$ at unitarity, exhibiting a good linearity. Plotted in the inset is an example case of ${N^{{\rm{ - 1/6}}}}{\rm{\ }}$ versus $t$, where the remaining atoms $N$ (normalized at $t = {\rm{0}}$) are counted and statistically averaged over three measurements. The vertical bars denote the standard error. Both the main figure and the inset yield nearly the same slope.

**Figure 3.**
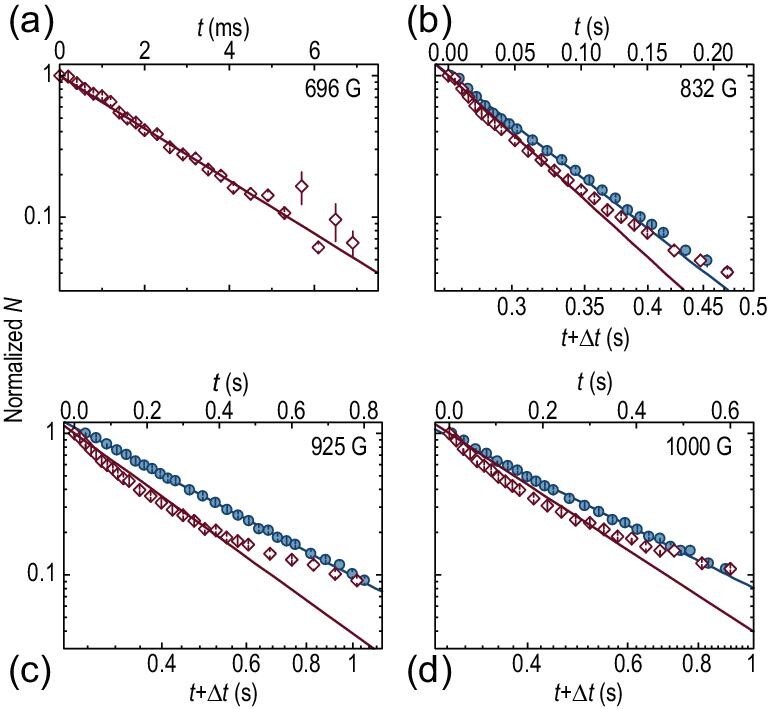
Optical molecular spectroscopy in the BCS-BEC crossover. Shown is the atom number $N$, normalized at $t = {\rm{0}}$, measured at 696, 832, 925 and 1000 G, as a function of $t$ on semi-log (red open diamonds, top axes) and log-log (blue filled circles, bottom axes) scales, with a laser power of (a) 20 $\mu {\rm{W}}$, (b) 60 $\mu {\rm{W}}$, (c) 120 $\mu {\rm{W}}$ and (d) 360 $\mu $W, respectively. Error bars represent one sigma standard error. The straight lines are exponential (a–d, red) and power-law (b–d, blue) fits, respectively, which yields $\alpha = {\rm{1}}{\rm{.68}}$ at 925 G and 2.10 at 1000 G.

Next, we measure ${Z_{{\rm{cc}}}}$ in the unitary and BCS regimes with the calibrated ${\rm{\Omega }}_{\rm{m}}^2/{\gamma _{\rm{m}}}$. At unitarity, $\alpha = {\rm{1/2}}$, and thus $N{( t )^{{\rm{ - 1/6}}}} \propto t + \Delta t$, with slope ${\rho ^{{\rm{ - 1/6}}}} = \beta /{\rm{6}} \propto \eta $. The ${{1/e}}$ decay time of the molecules is carefully chosen to be ∼60 ms, which is much longer than the estimated equilibration time of the dressed molecules [32]. The inset of Fig. 2 shows as an example ${N^{{\rm{ - 1/6}}}}$ versus ${{t}}$. The good agreement between the data and the linear fitting demonstrates the validity of the theoretical model. The fitted slope yields ${\rm{\eta }} = {\rm{0}}{\rm{.070}}( {\rm{1}} ){\rm{\ }}{{\rm{K}}^{{\rm{ - 1/2}}}}$, which is very close to the theory value of 0.066 ${{\rm{K}}^{{\rm{ - 1/2}}}}$ at $T = 0$. For comparison, exponential fitting clearly fails, as shown in Fig. 3(b) (open red diamonds), despite the fact that the change in ${Z_{{\rm{cc}}}}$ is only ∼30% during the molecular probing. We further perform a series of measurements of ${{\rm{Z}}_{{\rm{cc}}}}$ with varying initial ${T_{\rm{F}}}$, as plotted in Fig. 2, which exhibit a good proportionality between ${Z_{{\rm{cc}}}}$ and $\sqrt {{T_{\rm{F}}}} $, with a coefficient of 0.074(12)${\rm{\ }}{{\rm{K}}^{ - 1/2}}$, in quantitative agreement with a single set of probing data in the inset. In contrast, the measurements and analysis in ref. [23] allow only one value of ${Z_{{\rm{cc}}}}$ for each interaction strength.

With the same procedure, we probe ${Z_{{\rm{cc}}}}$ on the BCS side for $B =$ 850–1000 G. In these measurements, to eliminate the potential non-equilibrium effects caused by magnetic field ramping, the Fermi gas is evaporatively cooled at the same field $B$ for molecular probing. At unitarity, the system temperature can be determined by fitting the *in-situ* density distribution with the known equation of state (EoS) [33]. Unfortunately, we cannot quantitatively determine the temperature at higher magnetic fields, due to the lack of reliable knowledge of the EoS in the crossover region. Nevertheless, a roughly linear increase in temperature with the magnetic field could be inferred from the observed cloud size change through time-of-flight measurement [34]. We attribute the slight increase of temperature to the decrease of elastic scattering rate in the BCS regime. The rapid decrease of ${Z_{{\rm{cc}}}}$ with $B$ leads to a significant increase of the 1/*e* depletion time. To suppress the influence of background loss, we increase the laser power gradually from 60 $\mu {\rm{W}}$ to 360 $\mu {\rm{W}}$ to maintain an approximately identical ‘decay constant’ of ∼200 ms throughout the whole BCS regime. In Fig. 3, we plot $N$ (normalized at $t = 0$) as a function of ${{t}}$ for *B* = 696, 832, 925 and 1000 G, which spans from the BEC, the unitary, to the BCS regimes. While the exponential decay function fits well with the data at 696 G in the BEC regime (Fig. 3(a)), there is a progressively increasing systematic deviation as the field increases (red open diamonds). The failure of the exponential fitting (semi-log scales, top axes) becomes obvious in the unitary and BCS regimes. In contrast, the power-law fitting with increasing exponent works well for the unitary and BCS cases, as manifested by the good straight fitting lines in log-log scales (blue solid circles, bottom axes) in Fig. 3(b)–(d). These results provide direct evidence of the many-body effects in ${Z_{{\rm{cc}}}}$.

**Figure 4.**
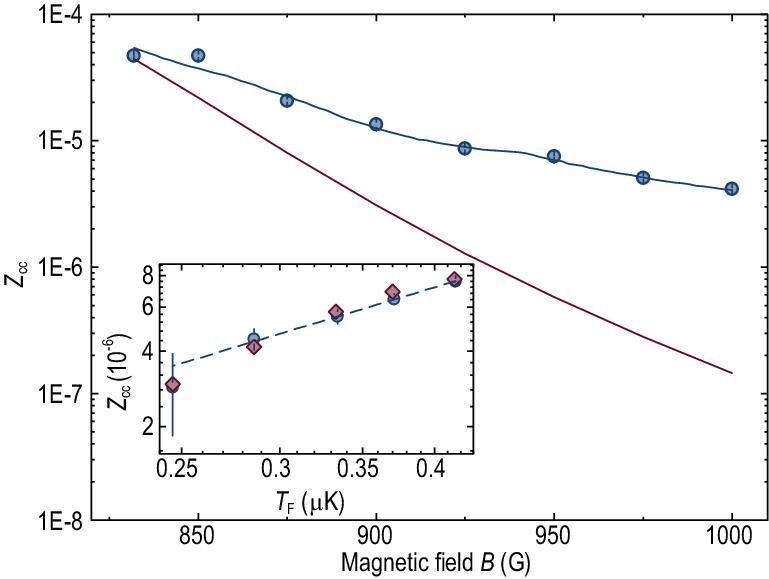
Measured closed-channel fraction ${Z_{{\rm{cc}}}}$ as a function of magnetic field for ${T_{\rm{F}}} = {\rm{0}}{\rm{.45\ }}\mu $K between experiment (blue solid circles) and theory at $T = {\rm{0}}$ (red curve) and at linearly varying $T/{T_{\rm{F}}}$ from 0.034 at 832 G to 0.056 at 1000 G (blue curve). Shown in the inset is ${Z_{{\rm{cc}}}}$ (blue solid circles) versus ${T_{\rm{F}}}$ at 925 G at low $T$ in log-log scale. The blue dashed line is a power-law fit with exponent $\alpha = {\rm{1}}{\rm{.48(8)}}$. For comparison, also plotted are the theoretical values (red diamonds) calculated at $T/{T_{\rm{F}}}$, linearly varying between 0.046 at ${T_{\rm{F}}} = {\rm{0}}{\rm{.416\ }}\mu $K and 0.049 at ${T_{\rm{F}}} = {\rm{0}}{\rm{.246\ }}\mu $K.

In Fig. 4, we compare the experimental and theoretical ${Z_{{\rm{cc}}}}$ values for ${T_{\rm{F}}} = {\rm{0}}{\rm{.45\ }}\mu {\rm{K}}$ on the BCS side of the FR. In the vicinity of unitarity, our experimental results are in good agreement with the many-body theoretical predictions calculated at $T = 0$. However, as the field increases, a progressively increasing departure between theory and experiment is found (see red curve in Fig. 4), which has also been noticed in ref. [16]. This is because in the BCS regime, the dispersion of finite momentum closed-channel molecules becomes significantly softened such that the population of non-condensed molecules at the trap edge becomes dramatically enhanced at finite ${\rm{T}}$. Thus, it is crucial to treat the thermal contributions of the closed-channel molecules properly, especially outside the superfluid core, in the BCS regime (see Supplementary Data for theoretical details). With theoretical improvements, semi-quantitative agreement between theory and experiment has been achieved, by assuming a finite but reasonable temperature (blue solid curve, $T/{T_{\rm{F}}}$ linearly varying from 0.034 at 832 G to 0.056 at 1000 G).

Presented in the inset of Fig. 4 is the measured ${Z_{{\rm{cc}}}}$ versus ${T_{\rm{F}}}$ at 925 G at low $T$ in log-log scale. The power-law fit (blue dashed line) yields an exponent $\alpha = {\rm{1}}{\rm{.48(8)}}$, which is consistent with that obtained from Fig. 3(c), but much larger than 1/2 at unitarity (there seems to be a slight curvature that agrees with the theory curve in Fig. S1). For comparison, also plotted are the theoretically calculated values (red diamondss) with $T/{T_{\rm{F}}}$ linearly varying between 0.046 at ${T_{\rm{F}}} = {\rm{0}}{\rm{.416\ }}\mu $K and 0.049 at ${T_{\rm{F}}} = {\rm{0}}{\rm{.246\ }}\mu $K, which exhibits a (semi-)quantitative agreement with the experiment. This slight temperature variation of $T/{T_{\rm{F}}}$ is reasonable since $T/{T_{\rm{F}}}$ was in fact slightly higher at lower ${T_{\rm{F}}}$.

Finally, we point out that, in a two-channel model, the density (or equivalently, ${T_{\rm{F}}}$) provides an extra dimension to the system. More specifically, two Fermi gases with different ${T_{\rm{F}}}$ are no longer mathematically equivalent, even if they share the same ${\rm{1}}/{k_{\rm{F}}}a$ and $T/{T_{\rm{F}}}$. This will inevitably lead to violation, albeit small, of the universality hypothesis of a unitary Fermi gas [35–37], which is based on a one-channel assumption. At the same time, over all BCS-BEC crossover regimes it is predicted that ${Z_{{\rm{cc}}}} \propto {{\rm{\Delta }}^{\rm{2}}}$ roughly for given ${T_{\rm{F}}}$ and interaction strength [16]. Furthermore, ${Z_{{\rm{cc}}}}$ also exhibits important $T$ dependence [16]. Hence measurement of condensed and non-condensed closed-channel fractions as a function of $T$ may disclose how the order parameter and the pseudo-gap evolve with temperature.

Note that we do not extract the (trap-averaged) Tan's contact [18] since its precise value is not yet available for comparison as a function of $B$ and $T$, especially in a trap.

## CONCLUSION

In summary, we have measured the ${Z_{{\rm{cc}}}}$ of interacting Fermi gases of ^6^Li in a trap as a function of ${T_F}$ and $B$. Away from the deep-BEC regime, the fraction ${Z_{{\rm{cc}}}}$ exhibits clear dependence on Fermi temperature ${T_{\rm{F}}}$, unraveling the important many-body interaction effect. In particular, we obtain ${Z_{{\rm{cc}}}} = \eta \sqrt {{T_{\rm{F}}}} $ at unitarity, with $\eta = {\rm{0}}{\rm{.074}}( {{\rm{12}}} ){\rm{\ }}{{\rm{K}}^{{\rm{ - 1/2}}}}$, in quantitative agreement with the theory. It would be interesting to perform a precision test of the universality hypothesis, investigate the temperature evolution of the condensed and non-condensed part of the closed-channel fraction, and test against different BCS-BEC crossover theories in the future.

## Supplementary Material

nwab226_Supplemental_File
