# Supplementary material for: Observation of the density dependence of the closed-channel fraction of a 6Li superfluid
Source: Natl Sci Rev. 2021 Dec 23;9(10):nwab226. doi: 10.1093/nsr/nwab226 (PMC9665308; doi:10.1093/nsr/nwab226)
Supplement: nwab226_Supplemental_File [file nwab226_supplemental_file.pdf]

Supplementary Data for
Observation of the density dependence of the closed-channel fraction of a ^{6}Li superfluid

Xiang-Pei Liu[1,2,3,*], Xing-Can Yao[1,2,3,*], Hao-Ze Chen[1,2,3], Xiao-Qiong Wang[1,2,3], Yu-Xuan Wang[1,2,3], Yu-Ao Chen[1,2,3,†], Qijin Chen[1,2,3,‡], K. Levin[4], and Jian-Wei Pan[1,2,3§]

[1] *Hefei National Laboratory for Physical Sciences at the Microscale and Department of Modern Physics, University of Science and Technology of China, Hefei 230026, China*

[2] *Shanghai Branch, CAS Center for Excellence in Quantum Information and Quantum Physics, University of Science and Technology of China, Shanghai 201315, China*

[3] *Shanghai Research Center for Quantum Sciences, Shanghai 201315, China and*

[4] *James Franck Institute, University of Chicago, Chicago, Illinois 60637, USA*

CLOSED CHANNEL FRACTION Z_{cc} AS A FUNCTION OF T_F IN THE BCS REGIME

According to the two-channel model in Ref. [1], in the homogeneous case, the total closed-channel molecule density $n_b^\mathrm{tot} = Z_g\tilde{\Delta}^2$ so that the closed channel fraction $Z_{cc} = 2n_b^\mathrm{tot}/n \propto \tilde{\Delta}^2/E_\mathrm{F}^{3/2} = (\tilde{\Delta}/E_\mathrm{F})^2 E_\mathrm{F}^{1/2}$, where the coefficient Z_g is roughly independent of density. At unitarity, the ratio $\tilde{\Delta}/E_\mathrm{F}$ is *essentially* independent of density in the case of a *wide* resonance, and thus one has the relation $Z_{cc} \propto \sqrt{T_\mathrm{F}}$. However, in the BCS regime, $\tilde{\Delta}/E_\mathrm{F} \propto e^{\pi/2k_\mathrm{F}a}$ so that $Z_{cc} \propto \sqrt{T_\mathrm{F}}e^{\pi/k_\mathrm{F}a}$ has a dominantly exponential dependence on $1/k_\mathrm{F}$. In a trap, the density dependence of closed-channel fraction will be modified. Nevertheless, at unitarity, for which $1/k_\mathrm{F}a = 0$ holds for the entire trap, the proportionality to $\sqrt{T_\mathrm{F}}$ still holds albeit with a different coefficient, as derived explicitly using a local density approximation in Ref. [1].

In the BCS regime, the local interaction parameter $1/k_\mathrm{F}a$ becomes non-uniform in a trap, so that the dependence of the closed-channel fraction Z_{cc} as a function of T_F can only be calculated numerically. We need to find out the right dependence in order to fit properly the experimental data of the number of remaining atoms versus probing time during the pumping process.

Figure S1 shows the closed-channel fraction Z_{cc} as a function of T_F in the unitary and BCS regimes for magnetic field $B = 832$ G, 900 G and 1000 G, which is plotted in a log-log scale. The curve at unitarity (832.18 G) is a straight line with a slope of 1/2. At unitarity, a linear plot of Z_{cc} vs $T_\mathrm{F}^{1/2}$ yields a straight line, $Z_{cc} = \eta T_\mathrm{F}^{1/2}$, with slope $\eta = 0.066$ K$^{-1/2}$, as shown in Fig. 3 of Ref. [1]. In the BCS regime, the slope of the curves increases as T_F decreases. Nevertheless, within the range of $T_\mathrm{F} = 0.2 - 0.6$ μK for our experimental data (yellow shaded area), the curves can be approximated by power laws, with a power-law exponent larger than $1/2$.

In Fig. S2, we present the measured atom number as a function of probe time in a linear scale, for different magnetic field strength ranging from BEC to BCS regimes. The solid lines in (b)-(d) are power law fits following Eq. (2) in the main text. The dashed lines in all panels are exponential fittings. While the failure of the exponential fitting starts to develop at unitarity, it becomes obvious in the BCS cases of 925 G and 1000 G.

IMPROVED TREATMENT OF THE CLOSED-CHANNEL POPULATION AT FINITE TEMPERATURES

The comparison between theory and experiment in this work was mostly based on a zero temperature calculation. This was fine except in the BCS regime, where we find it necessary to use an improved treatment that takes care of the thermal populations in the closed channel at finite temperatures, especially those outside the superfluid core and thus in the normal pseudogapped state.

In our two-channel model [3], the propagator $D(Q)$ of the

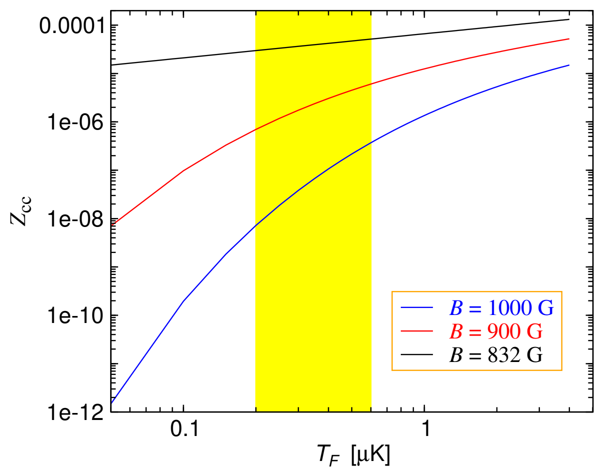

Figure S1. Log-log plot of the closed-channel fraction Z_{cc} as a function of T_F in the unitary and BCS regimes at $T = 0$. The yellow shade area corresponds to the range of our experimental data. This is essentially re-plotted from Fig. 3 of Ref. [1], but with the unitary data recalculated at 832 G with the most up-to-date resonance parameters from Ref. [2].

closed-channel molecules is given by

$$D(Q) \equiv \frac{1}{i\Omega_l - \epsilon_q^{\rm mb} - \nu + 2\mu - \Sigma_{\rm B}(Q)}\,. \quad \text{(S1)}$$

Here the molecular self energy $\Sigma_{\rm B}(Q)$ can be written as

$$\Sigma_{\rm B}(Q) = -g^2\chi(Q)/[1+U\chi(Q)]\,, \quad \text{(S2)}$$

where $\chi(Q) = \sum_K G_0(Q-K)G(K)\varphi^2_{\mathbf{k}}$ is the pair susceptibility, μ is the fermionic chemical potential, ν the magnetic detuning of the closed-channel molecules, $\epsilon^{\rm mb}_{\mathbf{q}} \equiv q^2/4m$ the bare kinetic energy of closed-channel molecules, U the open-channel inter-atomic interaction, and g the cross-channel coupling. This RPA-like self energy arises from interactions between the molecular bosons and the fermion pairs. See Ref. [3] for details. As usual, we set $\hbar = 1$, $k_{\rm B} = 1$, and use a four-vector notation, with $K \equiv (\mathbf{k}, i\omega_n)$, $Q \equiv (\mathbf{q}, i\Omega_l)$, $\sum_K \equiv T\sum_{\mathbf{k}}\sum_n$, $\sum_K \equiv T\sum_{\mathbf{k}}\sum_l$, where $\omega_n = (2n+1)\pi T$ and $\Omega_l = 2l\pi T$ are odd and even Matsubara frequencies, respectively. A large momentum cutoff k_0 is assumed in the symmetry factor $\varphi^2_{\mathbf{k}} = e^{-k^2/k_0^2}$, which is appropriate for a short range s-wave interaction. In numerical calculations shown in this section, we take $k_0/k_{\rm F} = 100$ at $T_{\rm F} = 1\mu$K, which is roughly given by the ratio of the inter-atomic spacing over the range of the Van der Waals potential. Note that this is indeed in the dilute regime, for which one may take safely the $k_0 \to +\infty$ limit.

Without repeating the details, we present in Fig. S3 a typical behavior of the real and imaginary part of propagator $D(Q)$. The curves were calculated for a homogeneous gas of ^{6}Li at $T_{\rm F} = 0.6\mu$K and $T = T_c = 0.2006T_{\rm F}$, using the resonance parameters given in Ref. [4]. This yields $\nu = 75560E_{\rm F}$ at $1/k_{\rm F}a = -0.5$, for the broad Feshbach resonance at 834.1 G.

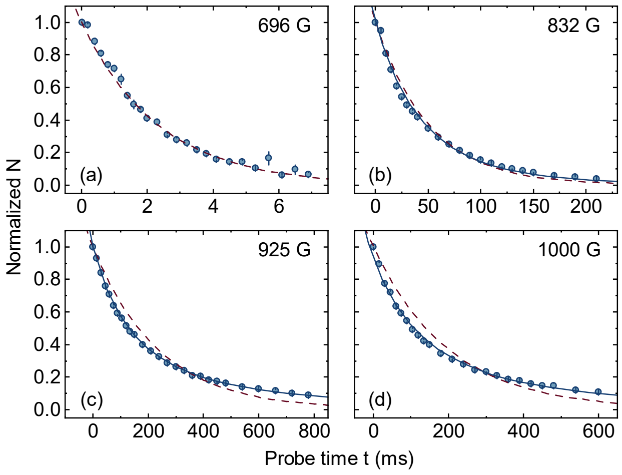

Figure S2. Atom number, normalized at $t = 0$, measured at 696, 832, 925 and 1000 G, as a function of t, plotted in linear scale, with laser power of (a) 20 μW, (b) 60 μW, (c) 120 μW , and (d) 360 μ W, respectively. The solid and dashed lines are power law and exponential fittings, respectively.

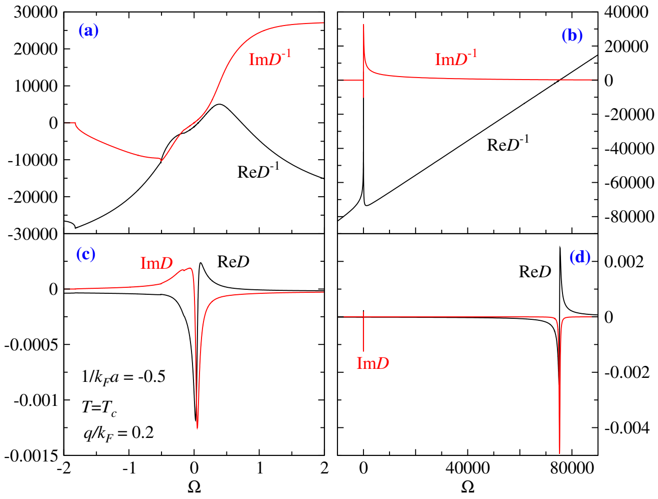

Figure S3. Typical behavior of the (inverse) propagator of closed-channel molecules for a homogeneous gas of ^{6}Li with a broad Feshbach resonance. Parameters are: $T_{\rm F} = 0.6\mu$K, $1/k_{\rm F}a = -0.5$, $T = T_c = 0.2006T_{\rm F}$, $k_0/k_{\rm F} = 100$, $U = -0.42E_{\rm F}/k^3_{\rm F}$, $g = 40.6E_{\rm F}/k_{\rm F}^{3/2}$, $\nu = 75560E_{\rm F}$, and $q = 0.2k_{\rm F}$. The spectral peak at low frequency in panels (c) and (d) becomes much sharper as q decreases. $E_{\rm F}$ and $k_{\rm F}$ are the units for energy and momentum, respectively, here and below.

Shown in Fig. S3 are the inverse molecule propagator $D^{-1}(\Omega)$ (top row) and the propagator $D(\Omega)$ (bottom row) in the real frequency axis for $q = 0.2k_{\rm F}$, in the low frequency range (left column) and a large frequency range (right column). The vast separation between the zeros of ReD^{-1} in panel (b) reflects the huge Feshbach resonance width, as indicated by the large value of g. The overall structure of D^{-1} and D in panels (a) and (c) mimics that of the hybridized T matrix, $t(Q)$. As one can see from panel (d), the spectral weight of the closed channel molecules are located almost 100% under the unoccupied peak at $\Omega/E_{\rm F} \approx 75560$ (red curve). The integrated spectral weight at low frequencies in panel (c) accounts for only a tiny fraction.

To calculate the closed-channel fraction accurately, one needs to evaluate the closed-channel population $n_{\rm b} = -\sum_Q D(Q)$ in a proper way, especially the thermal populations with $Q \neq 0$. To avoid extremely demanding numerics, one can Taylor-expand the inverse propagator $D^{-1}(Q)$ and inverse T matrix $t^{-1}(Q)$ in terms of Ω and $\mathbf{q}$. For $t^{-1}(Q)$, we obtain

$$\begin{aligned} t^{-1}(Q) &\approx Z_1(i\Omega_l)^2 + Z_0(i\Omega_l) - \xi^2\mathbf{q}^2 + t^{-1}(0) \\ &= Z_1(i\Omega_l)^2 + Z_0(i\Omega_l - \Omega_{\mathbf{q}}), \end{aligned} \quad \text{(S3)}$$

where $\Omega_{\mathbf{q}} = q^2/2M^* - \mu_{\rm pair}$, and $1/2M^* \equiv \xi^2/Z_0$, with the effective pair chemical potential $\mu_{\rm pair} = t^{-1}(0)/Z_0 = 0$ in the superfluid phase.

The pseudogap $\Delta_{\rm pg}$ is related to the density of pairs, via

$$\Delta^2_{\rm pg} = \sum_{\mathbf{q}} \frac{b(\tilde\Omega_{\mathbf{q}})}{Z_0\sqrt{1+4\dfrac{Z_1}{Z_0}\Omega_{\mathbf{q}}}}\,, \quad \text{(S4)}$$

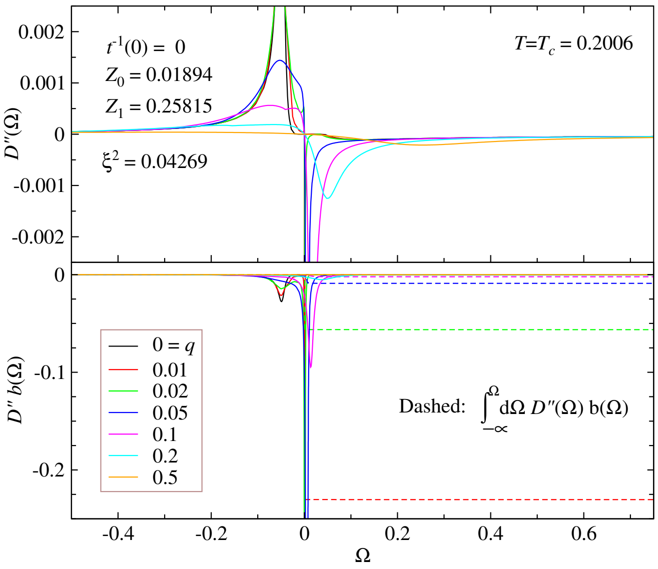

Figure S4. Behavior of $D''(\Omega)$ and $D''(\Omega)b(\Omega)$ (solid lines) and the spectral integral (dashed lines) as a function of Ω at T_c for a series of q. Other interaction parameters are the same as in Fig. S3. The units for momentum and energy are $k_{\rm F}$ and $E_{\rm F}$, respectively.

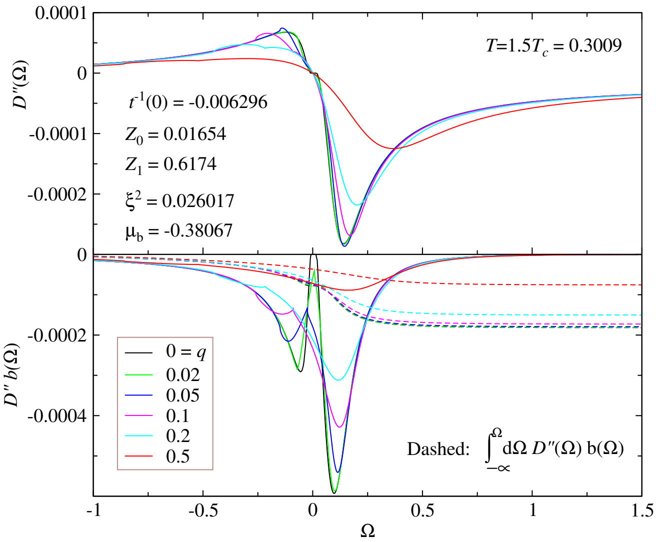

Figure S5. Plot similar to Fig. S4 but at $T = 1.5T_c$. $\mu_{\rm pair} = t^{-1}(0)/Z_0 = -0.3807E_{\rm F}$

where $b(x) = 1/(e^{x/T} - 1)$ is the Bose distribution function and $\tilde{\Omega}_{\mathbf{q}} = Z_0\{\sqrt{1 + 4Z_1\Omega_{\mathbf{q}}/Z_0} - 1\}/2Z_1$ is the pair dispersion.

In the unitary and BEC regime, especially in the superfluid phase, the contributions to the integral in Eq. (S4) mainly come from the small q regime. In this case, one can drop the Z_1 term, and thus has $t^{-1}(Q) \approx Z_0(i\Omega_l - \Omega_{\mathbf{q}})$ and $\Delta^2_{\rm pg} \approx \frac{1}{Z_0}\sum_{\mathbf{q}} b(\Omega_{\mathbf{q}})$.

The Taylor expansion of $D^{-1}(Q)$ follows closely this same procedure. Below T_c, both $D^{-1}(Q)$ and $t^{-1}(Q)$ yield the same dispersion, and thus they differ by an overall coefficient in the zero Q limit. Above T_c, the dispersions become different, and the effective chemical potential $\mu_{\rm b}$ of the closed-channel molecules will differ from that of the open-channel pairs. Nonetheless, this difference is small and we shall neglect it in the present treatment. As a result, in the long wavelength limit, one has

$$D(Q) \approx \frac{Z_{\rm b}}{i\Omega_l - \Omega_{\mathbf{q}}}\,, \tag{S5}$$

where the residue $Z_{\rm b} = Z_g/Z_0$ and $Z_g \equiv g^2/[(2\mu - \nu)U + g^2]^2$. (See Ref. [1] for details). One can then define the momentum distribution of closed-channel molecules as

$$n^0_{\rm b}(\mathbf{q}) \equiv -T\sum_l D(Q) = -\int_{\infty}^{\infty} \frac{d\Omega}{\pi} D''(\Omega)b(\Omega) \tag{S6}$$

$$= Z_{\rm b} b(\Omega_{\mathbf{q}})\,, \tag{S7}$$

where $D''(\Omega) \equiv {\rm Im}D(\Omega + i0^+, \mathbf{q})$. Equations (S5) and (S7) are the level of approximations used in Ref. [1]. At low T, the contributions are concentrated in the small q regime, where $\tilde{\Omega}_{\mathbf{q}} \approx \Omega_{\mathbf{q}}$, so that expression (S7) does not lead to significant error. However, in the BCS regime, the gap becomes small, and the Z_1 term in the above Taylor expansion quickly becomes important as Ω increases so that $\tilde{\Omega}_{\mathbf{q}}$ becomes much softer than $\Omega_{\mathbf{q}}$. This is especially true for $T > T_c$ where $\mu_{\rm pair} \neq 0$ so that the contributions to $n_{\rm b}$ are no longer dominated by the small q regime. Here we are mainly concerned with the treatment above T_c in the BCS regime. Now, taking into account the Z_1 term, one has

$$n^1_{\rm b}(\mathbf{q}) \equiv -T\sum_l D(Q) = Z_{\rm b}\frac{b(\tilde{\Omega}_{\mathbf{q}})}{\sqrt{1 + 4Z_1\Omega_{\mathbf{q}}/Z_0}}\,. \tag{S8}$$

Note that the Z_1 term has no effect on the condensate contribution, which is associated with $\Omega = 0$, $q = 0$ and $\mu_{\rm pair} = 0$.

Now we present our numerical analysis to show whether the Z_1 term yields a much improved numerical result for the closed-channel fraction.

Plotted in Figs. S4 and S5 are $D''(\Omega)$ and $D''(\Omega)b(\Omega)$ (solid lines) as well as the spectral integral with respect to Ω (dashed lines) on the real frequency axis, for $T = T_c$ and $T = 1.5T_c$, respectively. Other interaction parameters are the same as in Fig. S3. The propagator $D(Q)$ was calculated based on Eqs. (S1) and (S2). Curves for a series of momentum q are presented. For both temperatures, the spectral weight resides mainly inside the peak at positive frequencies. However, at T_c (Fig. S4), the main contribution, after multiplying with the Bose distribution function $b(\Omega)$, comes from the sharp spectral peak at positive frequencies, as can be seen from the step-like jump in the integral across this peak. At the same time, the integral decreases rapidly as q increases, so that the main contribution indeed comes from the small q regime and thus Eq. (S7) is a good approximation. In contrast, above T_c (Fig. S5), a substantial contribution actually comes from the negative frequency regime, as shown by the continuously rising (magnitude of the) integrals (dashed lines). The clustering of the dashed lines at small q indicates that the contribution

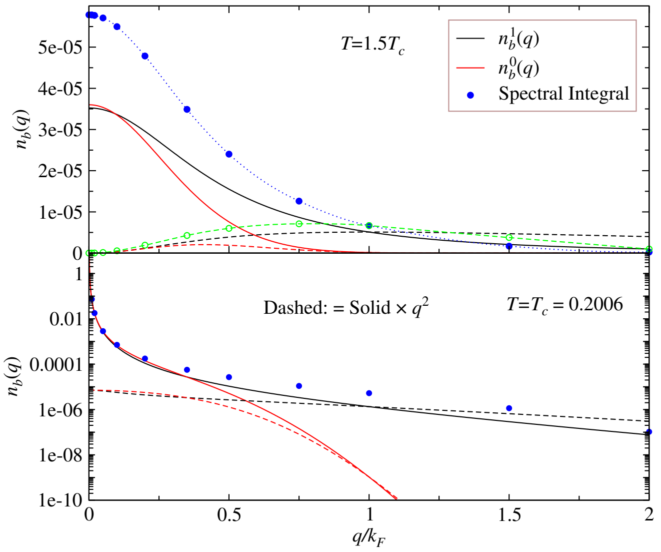

Figure S6. Comparison between $n_\mathrm{b}(q)$ obtained from spectral integrals (blue solid circles) with the approximated expressions $n^0_\mathrm{b}(q)$ (red solid) and $n^1_\mathrm{b}(q)$ (black solid lines) for both $T = T_c$ and $T = 1.5T_c$. The color coded dashed lines are obtained by multiplying corresponding solid lines with q^2. And the green dashed line is given by blue dotted line times q^2 in the upper panel. One concludes that $n^1_\mathrm{b}(q)$ is a much better quantitative approximation for the spectral integrated $n_\mathrm{b}(q)$.

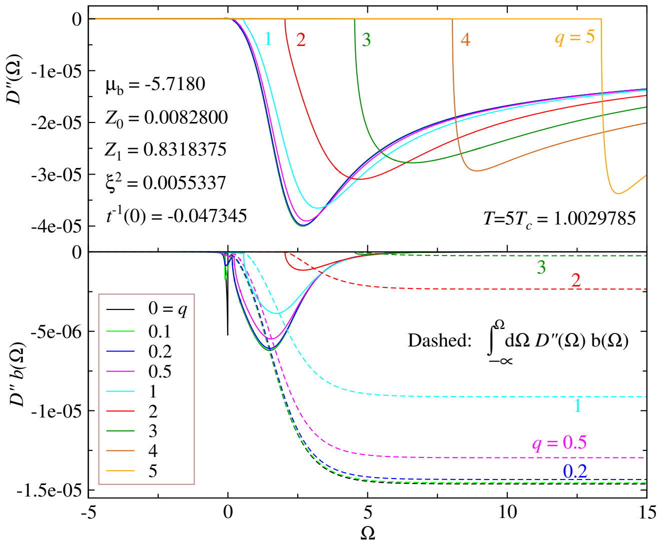

Figure S7. Plot similar to Fig. S4 but at $T = 5T_c$. The spectral weight for $\Omega < 0$ becomes very small and extends to large $\Omega > 0$ due to high T. Here $\mu_\mathrm{pair} \approx -5.72E_\mathrm{F}$.

to n_b is no longer dominated by the small q regime. In other words, Eq. (S7) is *no longer* a good approximation above T_c in this case.

Next, we plot in Fig. S6 the fully numerically obtained $n_\mathrm{b}(q)$ via Eq. (S6) (blue solid circles), as given by the spectral integrals in Figs. S4 and S5, and compare with the approximated result from Eqs. (S7) (red solid) and (S6) (black solid lines). The dashed lines are obtained by multiplying $n_\mathrm{b}(q)$ with the phase space factor q^2. To accommodate the fast decreasing $n^0_\mathrm{b}(q)$ versus q at T_c (lower panel), a logarithmic vertical scale has been used. At T_c, the small q regime clearly makes a major contribution. In contrast, above T_c (top panel), the small q regime (under the red dashed curve) only accounts for a small fraction, and thus the closed channel population calculated based on Eq. (S7) will lead to a serious underestimate. In both cases shown in Fig. S6, the expression for $n^1_\mathrm{b}(q)$, represented by the black solid curves, seems to be a good approximation for the result obtained from spectral integrals, with only a small underestimate.

To see how the comparison evolves with temperature, we repeated the same calculation at different temperatures above T_c. Our results show that as T increases further, the fermionic chemical potential decreases and the Fermi surface shrinks, and the effective bosonic chemical potential decreases as well. Then the excitation of the closed channel molecules becomes highly gapped, so that the spectral contribution from the negative frequencies decreases and then disappears. Shown in Fig. S7 is the behavior of $D''(\Omega)$ and $D''(\Omega)b(\Omega)$ for $T/T_\mathrm{F} = 5$ and a series of molecular momentum q. In comparison with Figs. S4 and S5, the spectral weight in $D''(\Omega)$ at negative frequencies is already invisible in the plot. On the other hand, the higher temperature allows the spectral weight to extend to much larger frequencies on the positive side, where the inverse T-matrix expansion at $\Omega = 0$ is no longer expected to be a good approximation. Note that, at $T = 5T_c \approx T_\mathrm{F}$, $\mu \approx -0.018E_\mathrm{F} < 0$ and the Fermi surface disappears. At the same time, μ_pair is already a large negative number.

Shown in Fig. S8 is comparison between $n_\mathrm{b}(q)$ (top row) obtained from spectral integrals (blue solid circles) with the approximated expressions $n^0_\mathrm{b}(q)$ (red solid) and $n^1_\mathrm{b}(q)$ (black solid lines) for $T/T_c = 2$, 3, and 5, from left to right, as labeled. Presented in the bottom row is $q^2 n_\mathrm{b}(q)$, so that the area under the curves yields the density of closed-channel molecules [up to a factor $1/(2\pi^2)$]. The result reveals that, as T increases further, while the underestimate of $n^0_\mathrm{b}(q)$ for $n_\mathrm{b}(q)$ becomes more severe, $n^1_\mathrm{b}(q)$ also becomes more and more an overestimate.

From the comparison in Figs. S6 and S8, we conclude that the Z_1 term in the Taylor expansion of the inverse pair and closed-channel molecule propagators need to be retained above T_c in the BCS regime. In a trap geometry, this corresponds to the pseudogapped state outside the superfluid core near the trap edge. Inside the superfluid core, the effect of this Z_1 term is quantitatively negligible. As shown in Fig. 4 in the main text, the thermal contribution at the trap edge can make a dramatic increase in the closed-channel population than that calculated at zero T, when the Z_1 term is taken into account. However, towards the very trap edge, which corresponds to a small local density or a high local $T/T_\mathrm{F}(r)$, one needs to cut off the contribution when using $n^1_\mathrm{b}(q)$ as an approximation. Without invoking overly demanding numerics, by allowing a reasonable finite temperature, one can bring the experimen-

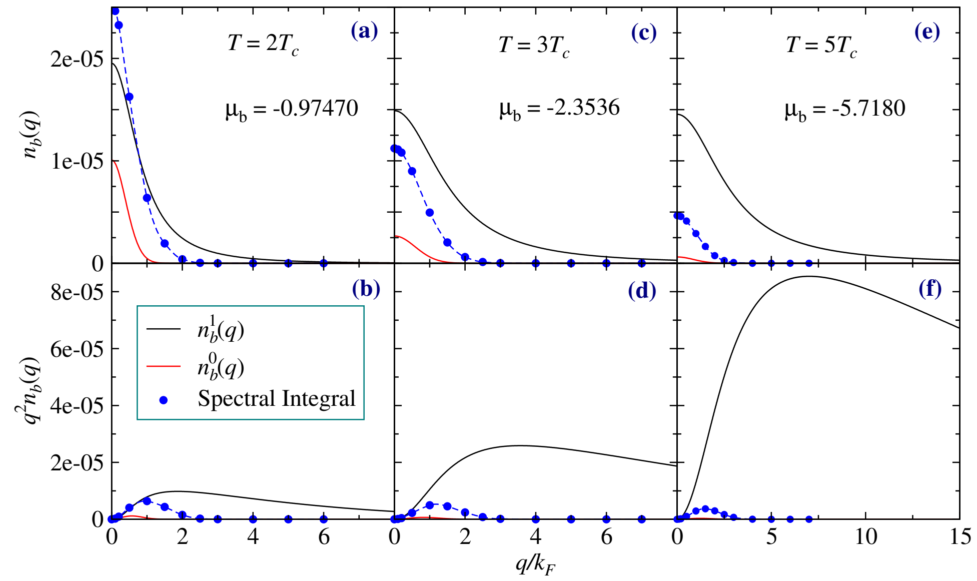

Figure S8. Comparison between $n_{\rm b}(q)$ obtained from spectral integrals (blue solid circles) with the approximated expressions $n_{\rm b}^0(q)$ (red solid) and $n_{\rm b}^1(q)$ (black solid lines) for $T = 2T_c$, $3T_c$ and $5T_c$, respectively, from left to right. The lower row is given by the top row multiplied by q^2. One can see that while $n_{\rm b}^1(q)$ is still a better approximation for the spectral integrated $n_{\rm b}(q)$, it becomes more of an overestimate as T increases further.

tal data and theoretical calculations into a good quantitative agreement.

Finally, for illustration purpose, we show an example of the density profile of the closed-channel molecules in a trap, calculated using the expression $n_{\rm b}^1(q)$ for the momentum distribution of the closed-channel molecules. Plotted in Fig. S9 are the spatial profiles of various densities as well as the gaps and

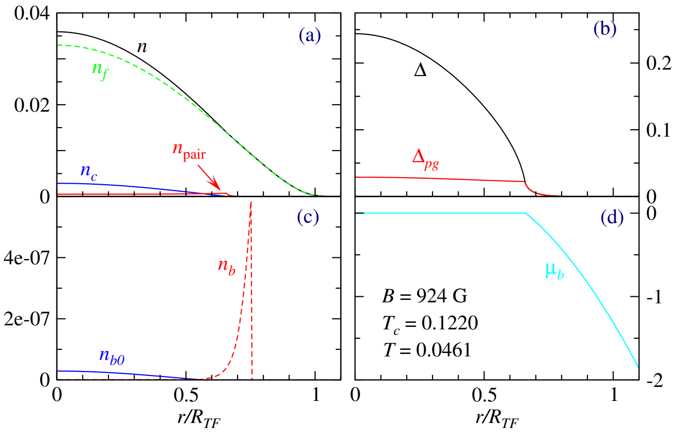

Figure S9. Spatial profiles of (a) the densities $n(r)$, fermions $n_{\rm f}(r) \equiv 2\sum_{\mathbf{k}} f(\xi_{\mathbf{k}})$, condense pairs $n_c = Z_0\tilde{\Delta}_{\rm sc}^2$, and noncondensed pairs $n_{\rm pair} = Z_0\Delta_{\rm pg}^2$, (b) total excitation gap Δ and pseudogap $\Delta_{\rm pg}$, (c) condensed closed channel molecules $n_{\rm b0} = Z_g\tilde{\Delta}_{\rm sc}^2$ and noncondensed closed-channel molecules $n_{\rm b}$, and (d) effective chemical potential $\mu_{\rm b}(\approx \mu_{\rm pair})$, as labeled, at 924 G at $T/T_c \approx 0.38$. Being too low, $n_{\rm b}$ is invisible on the vertical scale of panel (a). Here $f(x)$ is the Fermi distribution function, and $\xi_{\mathbf{k}} = k^2/2m - \mu$ is the bare atomic dispersion.

bosonic chemical potential calculated at 924 G ($1/k_{\rm F}a \approx -1$) and $T \approx 0.046T_{\rm F} \approx 0.38T_c$. This temperature was taken from Fig. 4 in the main text. The superfluid condensate core is located within $r_{\rm c1} \leq 0.56R_{\rm TF}$, where $R_{\rm TF}$ is the Thomas-Fermi radius. The condensed molecule density $n_{\rm b0}$ decreases with r and vanishes at the core edge. The noncondensed molecule density $n_{\rm b}(r)$ (red dashed line) is negligible inside the core, and starts to increase with radius outside the core. Note that at the core edge, the local density $n(r)$ is over half the density at the trap center, such that the local Fermi energy $E_{\rm F}(r)$ is still comparable to the global $E_{\rm F}$. Even at the cutoff radius $r_{\rm c2} \approx 0.75R_{\rm TF}$, one still has $n(r_{\rm c2}) \approx 0.26n(0)$, leading to $E_{\rm F}(r_{\rm c2}) \approx 0.40E_{\rm F}(0)$, not much smaller than the global $E_{\rm F}$. At this radius, $\mu_{\rm b} \approx -0.264E_{\rm F} \approx -0.65E_{\rm F}(r_{\rm c2})$. The absolute value of this $\mu_{\rm b}/E_{\rm F}(r_{\rm c2})$ is between that of Fig. S5 and of Fig. S9(a). We estimate that the corresponding local $T/T_c(r)$ is somewhere between 1.5 and 2. According to Fig. S8, $n_{\rm b}(r_{\rm c2})$ in Fig. S9(c) is somewhat an overestimate, and it is expected to decrease gradually with $r > r_{\rm c2}$. The cutoff radius $r_{\rm c2}$ is so determined that the local gap $\Delta(r_{\rm c2})$ drops below certain threshold, say, $1\times10^{-4}\Delta(0)$ for $r > r_{\rm c2}$). Since the experimental temperature measurement itself is not very accurate in the BCS regime, it suffices to use such a simplified estimate with a tunable temperature rather than taking a highly demanding fully numerical approach.

* X.-P. Liu and X.-C. Yao contributed equally to this work.
† yuaochen@ustc.edu.cn
‡ qchen@uchicago.edu

§ pan@ustc.edu.cn

[1] Q. Chen and K. Levin, "Population of closed-channel molecules in trapped Fermi gases with broad Feshbach resonances," Phys. Rev. Lett. **95**, 260406 (2005).

[2] G. Zürn, T. Lompe, A. N. Wenz, S. Jochim, P. S. Julienne, and J. M. Hutson, "Precise characterization of ^{6}Li Feshbach resonances using trap-sideband-resolved RF spectroscopy of weakly bound molecules," Phys. Rev. Lett. **110**, 135301 (2013).

[3] Q. Chen, J. Stajic, S. Tan, and K. Levin, "BCS–BEC crossover: From high temperature superconductors to ultracold superfluids," Phys. Rep. **412**, 1–88 (2005).

[4] M. Bartenstein, A. Altmeyer, S. Riedl, R. Geursen, S. Jochim, C. Chin, J. H. Denschlag, R. Grimm, A. Simoni, E. Tiesinga, C. J. Williams, and P. S. Julienne, "Precise Determination of ^{6}Li Cold Collision Parameters by Radio-Frequency Spectroscopy on Weakly Bound Molecules," Phys. Rev. Lett. **94**, 103201 (2005).
